# Evoked Potentials and Neuropsychological Tests Validate Positron Emission Topography (PET) Brain Metabolism in Cognitively Impaired Patients

**DOI:** 10.1371/journal.pone.0055398

**Published:** 2013-03-20

**Authors:** Eric R. Braverman, Kenneth Blum, Uma J. Damle, Mallory Kerner, Kristina Dushaj, Marlene Oscar-Berman

**Affiliations:** 1 Department of Clinical Neurology, PATH Foundation NY, New York, New York, United States of America; 2 Department of Psychiatry and McKnight Brain Institute, University of Florida, College of Medicine, Gainesville, Florida, United States of America; 3 Global Integrated Services Unit, University of Vermont, Center for Clinical and Translational Science, College of Medicine, Burlington, Vermont, United States of America; 4 Departments of Psychiatry, Neurology, and Anatomy and Neurobiology, Boston University School of Medicine and Boston VA Healthcare System, Boston, Massachusetts, United States of America; Banner Alzheimer's Institute, United States of America

## Abstract

Fluorodeoxyglucose (FDG) Positron Emission Topography (PET) brain hypometabolism (HM) correlates with diminished cognitive capacity and risk of developing dementia. However, because clinical utility of PET is limited by cost, we sought to determine whether a less costly electrophysiological measure, the P300 evoked potential, in combination with neuropsychological test performance, would validate PET HM in neuropsychiatric patients. We found that patients with amnestic and non-amnestic cognitive impairment and HM (n = 43) evidenced significantly reduced P300 amplitudes, delayed latencies, and neuropsychological deficits, compared to patients with normal brain metabolism (NM; n = 187). Data from patients with missing cognitive test scores (n = 57) were removed from the final sample, and logistic regression modeling was performed on the modified sample (n = 173, p = .000004). The logistic regression modeling, based on P300 and neuropsychological measures, was used to validate membership in the HM *vs.* NM groups. It showed classification validation in 13/25 HM subjects (52.0%) and in 125/148 NM subjects (84.5%), correlating with total classification accuracy of 79.8%. In this paper, abnormal P300 evoked potentials coupled with cognitive test impairment validates brain metabolism and mild/moderate cognitive impairment (MCI). To this end, we cautiously propose incorporating electrophysiological and neuropsychological assessments as cost-effective brain metabolism and MCI indicators in primary care. Final interpretation of these results must await required additional studies confirming these interesting results.

## Introduction

Dementia is the sixth leading cause of death in the United States, increasing in incidence and prevalence as the “baby boomer” generation ages along with longer life expectancies [1]. With the economic burden of dementia consistently rising [2], early identification of cognitive decline in primary care settings is imperative [3]. Decades of research involving brain electrophysiology have shown that delayed latency in the P300 brain wave (the positive spike in an EEG wave 300 ms after a stimulus) and a lower amplitude in the voltage of the P300 wave, occur in both normal aging, and even more so, in dementia [4]. However, little is known about the relation of electrophysiological parameters (P300), HM of the brain, and MCI/Alzheimer's disease (AD) markers such as tau proteins, C-reactive protein, and hippocampal atrophy [5], [6], [7]. If a patient diagnosed with clinical MCI is positive for these markers, prodromal AD should be considered. Magnetic Resonance Imaging or Angiogram (MRI, MRA) and PET are useful techniques that permit us to track abnormalities that may be markers of MCI or AD [8]–[11]. Both P300 and PET can detect early functional changes in MCI before anatomical damage becomes evident on MRI/MRA or neuropsychological profiles. There is also a paucity of information linking scores on the Mini-Mental State Examination (MMSE) [12] and brain HM in early cognitive decline [13], [14]. Finally, there are no studies to our knowledge that have evaluated the validation ability of three common assessment tools for revealing brain HM: Central Nervous System Vital Signs Memory Test (CNSM); Test of Variables of Attention (TOVA); and Wechsler Memory Scale-III (WMS). Our hypothesis is that evoked potentials and neuropsychological tests can validate PET brain metabolism and MCI, or early stages of Alzheimer's disease [15]. Therefore, the current retrospective study systematically examined the sensitivity and specificity of using P300, TOVA, and memory tests (WMS, CNSM, and MMSE) as early indicators of HM as measured by PET, in a cohort of patients with amnestic and non-amnestic cognitive impairments presenting to a large medical practice [16].

## Methods

### Participants

Of the more than 9,000 outpatients who visited a neuropsychiatric private practice group in Manhattan (1998–2009), 662 receiving a fluorodeoxyglucose (FDG) PET scan expressed interest in enrollment in the study, and signed written informed consent forms. The study sample was further refined by selecting patients (n = 240) with data available from P300 [17] visual and auditory evoked potentials, TOVA, WMS, MMSE, and CNSM. Subjects enrolled in the study performed testing on arrival and were advised not to take medications 24 hours prior to testing and were asked to refrain from caffeine, nicotine, and alcohol as well. Subjects did not undergo any Magnetic Resonance (e.g., MRI, MRA). Subjects indicated if they had depressive symptoms (n = 124) prior to the study and were evaluated for depression using the Millon Clinical Multiaxial Inventory-III and the Myers-Briggs Type Indicator (MBTI). Fifty-three percent (53.2%) of these subjects (n = 66) were found to be clinically depressed. Subjects were excluded (n = 10) if they showed evidence of structural brain lesions (e.g., brain tumors, strokes, encephalomalacia) on concomitant computed tomography (CT) brain scans, other neurologic disorders affecting brain functioning (e.g., multiple sclerosis, head trauma), serious systemic illnesses affecting cognitive functioning, or serious psychiatric disorders affecting cognitive functioning (e.g., schizophrenia, bipolar disorder, brain damage or injury).

The study sample was first divided into two groups: positive for PET HM (n = 43) or PET NM (n = 187), as interpreted by visual inspection of PET scans by radiologists. Hypometabolic subjects were arranged in groups according to brain regions where HM was detected: Groups 1 (parietal), Groups 2 (parietal+temporal/frontal), Groups 3 (frontal), Groups 4 (temporal), Groups 5 (focal), and Groups 6 (none). The HM subjects were further categorized as amnestic single domain (n = 1), amnestic multi domain (n = 19), non-amnestic single domain (n = 7), non-amnestic multi domain (n = 14), or no signs of MCI (n = 2). The NM subjects were also categorized as amnestic single domain (n = 3), amnestic multi domain (n = 27), non-amnestic single domain (n = 50), non-amnestic multi domain (n = 88), or no signs of MCI (n = 19).

### FDG PET Scans

The narrative reports from the neuroradiology group were divided into six groups of hypometabolism: parietal, parietal plus temporal/frontal, frontal, temporal, focal, or none. The original reading of the neuroradiology group was reconfirmed with visual inspection of the DICOM PET images.

The PET scans completed by a private neuroradiology group (MedScan) were conducted with either a whole-body or brain-specific high-resolution PET (Siemens/CTI ECAT HR+, with 4.6×4.6×4.2 mm NEMA; National Electrical Manufacturers Association) using FDG. Methodological details for scanning have been published [18]. Prior to PET imaging, a diagnostic quality CT scan of the brain was performed without intravenous contrast, and the patient's blood glucose level was assessed as being within normal limits. After the CT scan, 14–18 mCi of FDG was administered intravenously. PET scan imaging was performed approximately 50 minutes after the administration of the radioisotope. Forty-seven slices were obtained at approximately 3.3 mm thickness, covering the entire brain parenchyma from the base of the cerebellum to the vertex.

CDs of the DICOM image data of the PET scans were converted to Analyze format utilizing MRIcro [18], which also anonymized the images to which blinded IDs were assigned. The analyzed formatted images were then imported to Statistical Parametric Mapping [19], where they were reviewed to exclude any scans with significant movement artifact, or areas of hypometabolism related to structural brain disorders not evident in the chart review, but observable on CT scan.

### EEG, P300, and Evoked Potentials Data

The P300 potential was obtained using Lexicor and Cognitrace. Twenty electrodes were used (5 in frontal region, 2 frontal temporal, 3 occipital, 2 temporal, 2 temporal parietal, 3 parietal, and 3 along the central sulcus). The two machines were calibrated with repeat scans. Both Lexicor and Cognitrace use auditory stimuli of low and high beeps, and provide an output of latency and amplitude based on preprogrammed baselines based on age. The latency (in milliseconds) and voltage (in microvolts) from the waveform selected for analysis were calculated by the computer algorithm and documented in the patients' charts. All data were anonymized with confidential IDs matching those of the PET scans.

### Cognitive Tests for MCI

Data were also collected regarding patients' memory complaints. Memory complaint data were used to determine whether the patient met clinical criteria for MCI: (1) the patient is neither normal nor demented; (2) evidence of cognitive deterioration indicated by subjective report of decline by self and/or informant in conjunction with objective cognitive deficits, or objectively measured cognitive decline over time; (3) and activities of daily living are either intact or only minimally impaired (Table 1) [20]. The CNSM test was computer-administered, and the resultant scores were recorded. The MMSE and WMS Immediate Memory (IM) Index (combining Immediate Verbal with Immediate Non-Verbal Memory scores) were recorded as well as the Wechsler Working Memory (WM) Index Score. The TOVA was computer-administered, and the Omission, Commission, Response Time, and Variability scores were recorded. Clinical and cognitive interviews also were conducted, and based upon combined assessments, the patients were categorized into four MCI domains: amnestic single domain, amnestic multi domain, non-amnestic single domain, or non-amnestic multi domain.

**Table 1:** MCI Domain Assessment.

	Domain	Yes	No	For Staff Use – any under 10^th^ percentile
1	**Attention**deficits indicated by missing stop signs, jumping the gun, slow response time, or inconsistency in manner of response	□	□	TOVA
2	**Reaction Time**	□	□	CNSVS, TOVA
3	**Judgment**the ability to make good decisions	□	□	CNSVS, TOVA
4	**Learning Ability**understanding concepts or instructions and ability to reason	□	□	WMS, CNSVS, WAIS
5	**Delayed Recall**free (without assistance), cued (with assistance of stimulus or prompt), or serial (recall items/events in order in which they were learnt), ability to retrieve information a given time period after which it was learnt	□	□	WMS, CNSVS
6	**Linguistic Function**ability to communicate effectively	□	□	MMSE
7	**Verbal IQ**ability to analyze information and solve language based problems of a literary, logical, or social type; understanding relationships between language concepts and performing language analogies and comparisons	□	□	WAIS
8	**Performance IQ**ability to analyze and utilize visual information, such as drawing or completing pictures, manipulating blocks to build structures	□	□	WMS II
9	**Abstract IQ**ability to analyze information and apply knowledge in problem solving using theories, metaphors, or complex analogies; usually involves forming ideas about the nature of objects, ideas, and processes; problems are often visual and typically do not involve social ideas	□	□	GAMA
10	**Processing Speed**how quickly/efficiently the brain processes the information it receives; ability to think and learn quickly	□	□	CNSVS, P300
11	**Immediate Memory**a general change in ability to remember things, short lists, things from one second to the next, recent events, etc.	□	□	WMS, RANDT, MMSE
12	**General Cognitive Functioning**broadly, the ability to think about ideas, analyze situations, and solve problems	□	□	CNSVS

* MCI Patient Checklist.

### Statistical Analyses

Student's t-test was calculated between the HM group and the NM group, as well as between subjects with and without memory complaints, for P300 latency and amplitude, TOVA reaction time (RT), MMSE score, WMS IM, and CNSM.

It is noteworthy that TOVA RT measures continuous performance, which requires action, whereas P300 is an internal cerebral reaction performance test that does not require action. Consequently, the difference between P300 latency and TOVA RT correlates to the time difference between thought and action. Thus, we calculated two different scores using simple transformation mathematics. In one case, the difference between the P300 latency and TOVA RT (P300-TOVA Diff) was calculated, as well as the absolute value of this difference (Abs Val Diff). Moreover, to account for age differences in P300, a transformation of raw P300 latency was calculated as a difference score between the obtained latency and the predicted latency from the age adjustment [obtained latency−(300+age)], such that positive numbers reflect slower latencies and negative numbers reflect faster latencies (P300AgeDiff). Logistic regression was then utilized to classify patients into groups based on the neurophysiology and memory measures as predictors adjusted to the percent of the sample being predicted.

## Results

### Correlation of PET with Evoked Potentials and Neuropsychological Test Results

Characteristics of the study sample, consisting of 43 patients with HM and 187 patients with NM, are listed in Table 2. The HM group was significantly older (t = 3.52, p = .007) than the NM group. Table 2 also displays statistics for the memory scores and neuropsychological (P300) measures between the HM and NM groups. As expected, the HM group had lower scores than the NM group for the MMSE (t = −2.73, p = .01) and CNSM (t = −2.82, p = .0001). The WMS IM Index (e.g., Verbal+Visual) and WMS WM Index were not significantly different between the two groups. Using a cutoff of −1.5, SD subjects (as recommended by the test details are considered to be borderline at the −1.5 SD) with impaired WMS IM Index scores did not differ significantly from those with intact scores when compared with hypometabolic and normal metabolic groups.

**Table 2:** Means, SDs, and t-tests of HM *vs.* NM subjects.

	Hypometabolic n = 43	Normal n = 187	Significance
Age	59.8±19.3	54.1±12.8	0.007
P300 Latency	346.7±29.7	327.5±27.3	.00006
P300 Amplitude	3.5±2.4	4.7±2.3	.003
P300AgeDiff	−15.7±26.1	−26.6±26.5	.015
TOVA* Reaction Time (RT)	346.8±29.7	327.5±27.3	.00006
P300–TOVA RT	83.8±95.0	30.5±67.0	.002
Absolute Value of Difference of P300–TOVA RT	93.7±85.0	54.1±49.8	.009
MMSE*	26.3±5.1	28.7±1.9	.010
CNSM*	84.7±31.5	100.5±18.6	.000099

* TOVA = Test of Variables of Attention; MMSE = Mini-Mental State Exam; CNSM = Memory score from the CNS Vital Signs Test.

However, the comparison of NM/HM to non-impaired/impaired CNSM scores (−1.5SD) was significant (χ^2^ = 10.7, p = .003), with more HM patients presenting poor memory (n = 11/23) than those with NM (n = 19/156). Poor scores on the MMSE (−1.5SD) also differed significantly (χ^2^ = 5.54, p = .027) between HM patients (7/35) *vs.* NM (11/155).

Evoked potential and reaction time data for the P300 and the TOVA are also displayed in Table 2. The P300 latency was significantly longer (t = 4.10, p = .00006) and the amplitude significantly lower (t = 3.04, p = .007) in the HM group compared to the NM group. The TOVA RT was significantly longer (t = 3.82, p = .00006) in the HM group compared to the NM group. The difference score between the TOVA RT and P300 latency was significantly greater (t = 2.74, p<.002) in the HM than the NM group, as was the absolute value of this difference (AbsValDiff, t = 2.74, p = .009).

The presence of memory complaints also differentiated one memory variable and several neurophysiologic variables (Table 3). Patients with memory complaints had lower MMSE scores (t = −2.23, p = .027), but did not differ from those without memory complaints on the CNSM, WMS IM, or WMS WM Indexes. Patients with memory complaints were also older (t = 2.95, p = .003), showed prolonged latencies (t = 2.72, p = .007) and decreased amplitude (t = −2.83, p = .005) on the P300, and showed longer RTs on the TOVA (t = 2.72, p = .007). The difference was most marked between the TOVA RT and P300 latency (t = 5.41, p = .000001). Comparing the relationship of memory complaints to HM, 40 of the 43 HM patients (93%) complained of memory problems, whereas 102 of the 187 NM patients (54%) presented with memory complaints (χ^2^ = 21.9, p = .000001). Significant values were not found for P300AgeDiff, WMS indices, and CNSM indices.

**Table 3:** Means, SDs, and significance of subjects with and without memory complaints (Cx).

	With Memory Cx	Without Memory Cx	Significance
No. of subjects	142	88	N/A
Age	57.9±13.9	52.2±14.6	0.003
P300 latency	335.1±29.5	324.6±26.3	.007
P300 amplitude	4.1±2.4	5.0±2.3	.005
TOVA* Reaction Time (RT)	392.0±83.4	333.5±55.6	.007
P300–TOVA RT	58.5±78.6	9.2±56.9	.000001
Absolute Value of Difference of P300–TOVA RT	71.0±67.5	44.4±36.5	.0002
MMSE*	27.9±3.5	28.8±1.6	.027

* TOVA = Test of Variables of Attention; MMSE = Mini-Mental State Exam.

The presence or absence of memory complaints and HM was also examined by dividing the sample into four subgroups and performing one-way analyses of variance (ANOVA). The ANOVA of the P300 amplitude was significant (F = 4.46, p = .005), and *post hoc* analyses showed that the patients with memory complaints and HM showed significantly lower amplitudes than those without memory complaints and NM (p = .002). The analysis of AbsValDiff was significant (F = 6.97, p = .00017). *Post hoc* analyses showed the HM group with memory complaints had a significantly greater absolute value difference between TOVA and P300 (95.41 ms) than the NM subgroup with memory complaints (62.8 ms, p = .021) and the NM subgroup without memory complaints (43.5 ms, p = .00006). ANOVA was significant for the MMSE (F = 7.39, p = .0001) with HM patients with memory complaints scoring below NM patients with memory complaints (p = .0002) and NM patients without memory complaints (p = .0001). The ANOVA was also significant for CNSM (F = 6.10, p = .001); HM patients with memory complaints scored below NM patients both with (p = .0003) and without (p = .002) memory complaints.

Those patients missing TOVA, CNSM, and MMSE scores (n = 57) were removed from the final sample (n = 230) and logistic regression modeling was performed on the modified sample (n = 173). Logistic regression analysis was used to validate membership in the HM *vs.* NM groups using P300 latency (e.g., the difference score between the obtained latency and the predicted latency from the age adjustment [obtained latency−(300+age)]) and P300 amplitude, the absolute value of the difference score between the TOVA RT and P300 latency, and scores on the MMSE and CNSM. The best model was significant (F = 13.2, p = .000004) and retained the CNSM score (Wald = 6.41, p = .011) and the absolute value of the difference score between the TOVA RT and P300 latency (Wald = 4.88, p = .025). The MMSE and P300 amplitude were not retained as validators. The classification model correctly validated 13 of 25 HM subjects (52.0%) and 125 of 148 NM subjects (84.5%) for overall classification accuracy of 79.8%. We recognize that our model demonstrates high specificity with lower sensitivity. Because of the clinical interest in determining HM validating MCI or later dementia in patients with memory complaints, the HM group with memory complaints was compared to the NM group without memory complaints with Logistic Regression entering age, memory scores, and neurophysiologic measures as validators (same as above). This analysis was significant (F = 16.4, p = .000001) retaining CNSM (p = .011) and TOVA-P300 difference score (p = .001). The classification model was significant (χ^2^ = 28.8, p = .000001) and correctly classified 15 of the 28 HM patients (53.6%) with memory complaints and 72 of the 79 (91.1%) of the NM patients without memory complaints for an overall correct classification of 81.3%.

With regard to the HM subjects, and because of the small sample sizes in Groups 3 (frontal), 4 (temporal), 5 (focal), and 6 (none), only Groups 1 (parietal) and 2 (parietal+temporal/frontal) were compared. The majority of HM patients showed abnormalities in the parietal lobe (Table 4). Group 2 had significantly lower (p = .021) WMS IM scores (96.5, SD = 22.5) than Group 1 (112.75, SD = 19.6) as well as significantly lower scores (p = .047) on CNSM (74.4, SD = 31.5 *vs.* 101.6, SD = 22.3). The two groups did not differ on any of the P300 latency or amplitude variables. It is noteworthy that 66 participant subjects from the present cohort were diagnosed with depression. We found that 54 of these patients were classified as NM, while only 12 were classified as HM. We also found that 50 of these patients were classified as having amnestic MCI, while 16 were classified as having non-amnestic MCI. All depressed HM subjects (n = 12) have amnestic MCI.

**Table 4:** Brain regions affected in HM subjects.

Group Number	Brain Region of Hypometabolism	HM Subjects Affected
1	parietal	18
2	parietal+temporal/frontal	16
3	frontal	3
4	temporal	1
5	focal	8
6	none	5

* Subjects overlapped in categories.

As noted earlier, both HM and NM subjects were evaluated for signs of MCI according to four domains [21]; two HM subjects showed no signs of MCI. Of the HM subjects, 47% were amnestic, 49% non-amnestic and 4% showed no signs of MCI progression (Table 5). For convenience purposes, subjects were categorized into one table: HM/NM, exhibiting signs of MCI/no signs of MCI, and memory complaints/no memory complaints (Table 6).

**Table 5:** Amnestic *vs.* Nonamnestic in NM and HM patients.

	Normal Brain Metabolism (n = 187)	Hypometabolic (n = 43)
**Single Domain Amnestic**	1.6%	2.3%
**Multi Domain Amnestic**	***14.4%***	***44.2%*** *
**Single Domain Nonamnestic**	26.7%	16.3%
**Multi Domain Nonamnestic**	***47.1%***	***32.5%*** *
**Not Affected**	10.2%	4.6%

* The majority of hypometabolic patients were found to be multi domain according to the MCI Domain Assessment.

**Table 6:** Overall Classification of Subjects.

Categories	No. of Subjects
**Hypometabolic**	43
**Normal Brain Metabolism**	187
**Signs of MCI (amnestic+nonamnestic)**	209
**No Signs MCI (amnestic+nonamnestic)**	21
**Memory Complaints**	142
**No Memory Complaints**	88

## Discussion

To reiterate, in the present study using FDG PET scans, we found significantly impaired P300 evoked potentials in those subjects with HM compared to those subjects with NM. The P300 latency was significantly longer and the amplitude was significantly lower in the HM compared to the NM group. While other reports have suggested the importance of event related potentials of the brain as a biomarker for decline in cognitive processes including MCI and AD [22]–[25], this is the first report that has used FDG PET scans to demonstrate a significant association between brain metabolism and event related potentials. We are encouraged that with additional confirmation of these results, our initial hypothesis will be borne out.

There have been many patterns used to diagnose MCI. Ferris [2] classified multiple stages, but the most appropriate classification system is the subdivision into amnestic single or multi domain and non-amnestic single or multi domain. This four category subdivision is accomplished through the following series of tests: attention; reaction time; judgment; learning ability; delayed recall; linguistic function; verbal IQ; performance IQ; abstract IQ; processing speed; immediate memory; and general cognitive functioning [26], [27]. Subjects who are positive in more than one of these areas are placed into regions, either single or multi domain. The annual conversion rate of MCI to dementia is approximately 4.2% in the general population [28].

In the present study, 76.7% of the HM patients were multi domain. The most common brain region affected in these patients was the parietal lobe (n = 18), affecting 41.8% of the HM group. According to Jacobs, et al. [29], parietal lobe activity characterizes early MCI. With a new concept of the regional spread of Alzheimer's or tauopathy, it is likely that some MCI patients progress with abnormalities in parietal regions, toward abnormalities in the temporal and frontal lobes, similar to a disease that progresses forward [28]. Disease begins with the loss of electrophysiological processing speed followed by voltage, thought to action gaps (e.g., TOVA, P300), and several other forms of cognitive domains. Once progression occurs to hypometabolic loss in the parietal lobes, it is simultaneously marked by demyelination, atrophy, and micro stroke, as seen on MRI scans [30]. This progression is unpredictable because once the process begins, it is extremely difficult to reverse even with the incorporation of new neurogenesis approaches (including serotonin-norepinephrine reuptake inhibitors, insulin-like growth factor 1, selective serotonin re-uptake inhibitors, fish oil, dehydroepiandrosterone, and a variety of other neuroendocrine and nutritional advances). The correlation and supplementation of this primary care data with MRI hippocampal volume is still ongoing [31].

The value of using MMSE as a screening tool to identify cognitive decline has been praised [32] and questioned [33]. Our lab suggested that TOVA also showed promise as a validator of early cognitive decline and/or dementia. Our previous research correlated TOVA abnormalities with impaired WMS scores of early dementia. Coupling of TOVA assessment findings with results of P300, MMSE, and WMS-III may allow for enhanced accuracy in the diagnosis and evaluation of the complex pathways of failing attention, memory, and cognition that lead to dementia [16]. In addition, Gualtieri and Johnson reported on the reliability and validity of the computerized neurocognitive test battery, CNS Vital Signs (CNSVS) [34]. Among the cognitive scores, the MMSE, TOVA, and CNSM scores were significantly lower in the HM compared to the NM group, thus supporting earlier reports [16], [32]. Comparing NM/HM to non-impaired/impaired CNSM was significant, with more HM patients presenting poor memory than those with NM.

Logistic regression modeling significantly validated membership in the HM group with memory complaints *vs.* the NM group without memory complaints (correctly classifying 81.3% of all patients) using the absolute value of a difference score calculated between each subject's TOVA RT and P300 latency, and scores on the CNSM. Based on our earlier work [17], it was not surprising to find that logistic regression modeling did not retain MMSE as a valuable validator in this study. This supports other studies in the literature that suggest it has limited predictive validity [17], [35]–[40].

We are cognizant of certain limitations in the present study (e.g., MCI/dementia not specifically evaluated for the current population) [41]. It is tempting to speculate that coupling both the impaired electrophysiological and neuropsychological parameters may provide a less expensive test than FDG PET to assist the primary care physician in diagnosing memory deficits. Thus, large independent studies including additional work coupling impaired P300, validated neuropsychological memory tests, and activities for daily living with FDG PET is encouraged. Another limitation of the current study is that the interpretation of FDG PET scans as hypometabolic or normal were made by visual inspection of the reconstructed scans instead of quantitative analysis of the actual voxel values, either globally or refined by a region of interest analysis. The current study may be extended to include such a quantitative analysis. Since brain volume decreases after age thirty at 0.2% per year [42], [43], another relative weakness of the study was excluding other imaging modalities such as PET beta amyloid tracers, VILIP-1, F-ML-10 apoptosis measures, MRI hippocampal volume, cortical thickness, demyelination, and micro ischemia, which can be combined to earlier detect pre-clinical states of MCI [44]–[46]. We are cognizant that the depressed subjects showing NM (n = 54) being treated with anti-depressants may have skewed the data because of medication-induced normalization of brain metabolism.

Importantly, the finding of a high specificity of the variables in validating HM is not surprising. However, while the sensitivity appears low by comparison to the specificity, we propose that the sensitivity remains beneficial: if indeed HM is validated in half of the patients, it is highly likely to be present and warrants further investigation for the presence of MCI or dementia. Forty-eight percent (48%) of the HM subjects were false negatives. It is important to utilize the PET scan more selectively, indicating that these 48% receive a PET scan. Fifty-two percent (52%) are ruled out because they do not require the PET scan for early diagnosis.

Results of the present study demonstrated for the first time that electrophysiological parameters (e.g., P300) coupled with neuropsychological measures (e.g., TOVA and CNSM) validate brain HM with clinically useful sensitivity and specificity. The P300 latency was significantly longer and the amplitude was significantly lower in the HM compared to the NM group, and these effects were retained after age correction. Neurocognitive measures (e.g., the MMSE [p = .01], TOVA [p = .00006], and CNSM [p = .0001] scores) were also significantly lower in the HM compared to the NM group. NM/HM compared to non-impaired/impaired CNSM were significant (p = .003), with more HM patients presenting poor memory than patients with NM.

It also appears that patients with single domain cognitive decline have less progression to dementia, followed by those who are multi-domain amnestic or non-amnestic, causing the use of electrophysiology to deteriorate [29], [47], [48]. As MCI atrophy sets in and advances to dementia, positive hypometabolic FDG PET scans are helpful indicators. Brain metabolism then creates a cognitive tipping point that is indicative of dementia or AD [31]. Our subjects, although incompletely characterized, had MCI by a series of neuropsychological measures. Current characterizations suggest that MCI needs greater stratification considering the many patients who already have features of dementia. Most patients with hypometabolic FDG PET scans do have early dementia, even if their current symptoms resemble only MCI [15].

MCI is heterogeneous - with electrophysiological decline, memory and attention failure, multiple domains of cognitive deterioration with and without losses of hippocampal volume, and cortical atrophy - steering patients from MCI into dementia. There are numerous clinical variants of MCI that are antecedents to dementia, where progression is altered dependent on different cognitive phenotypes (e.g., individuals who emphasize working memory *vs.* auditory memory, abstract IQ *vs.* emotional IQ) [49]. A similar progression occurs in heart disease based on risk factors (e.g., cholesterol [HDL/LDL], electrophysiological dysfunction, valve and coronary artery disease, hormonal and vascular factors) which may occur in any combination dependent on genetic predispositions or environmental factors. Although no one system is perfectly predictive, an in-office model has been implemented, where electrophysiological decline, particularly, delays of processing speeds when moving from thought to action (e.g., TOVA and P300) seem to be validating PET hypometabolism.

## Conclusions

A significant rise in AD is expected with a continuing demographic shift to a more elderly population. AD is predicted to increase from 4.5 million in 2000 to 13.2 million in 2050 as “baby boomers” age and life expectancy increases [50]. If primary care practices implement proper MCI checklists, P300, and TOVA testing done within an hour's time, physicians will be able to diagnose early MCI antecedents [51]–[55]. Sustaining our intellectual faculties with age may be possible with early diagnosis and treatment. These practices may also have economic advantages as a patient can receive the MCI domain assessment, electrophysiological markers and brain testing at a cost-effective price of $500, while PET scans still remain at an expensive price of $3000–6000 per patient. This proposal may aid in lessening the United States' $200 billion dementia burden by identifying high risk patients through multiple domains (e.g., P300 low voltage and slow speed and temporal differences between thought and action) [56]. These clinical implications may potentially impact the epidemic of dementia at a primary care level, similar to the ways an electrocardiography (ECG), cholesterol testing (HDL/LDL), and the echocardiogram lessened the cardiac burden worldwide.

Future work confirming this clinically relevant research may indeed provide sufficient evidence to suggest the incorporation of impaired electrophysiological and neuropsychological determinants as an efficient means for identifying and validating reduced brain metabolism and cognitive impairment in MCI care settings leading to an early hallmark identifier of patient progression to dementia. We must await further studies before any real interpretation can be garnished from this important preliminary study.
